# LncRNA KRT19P3 Is Involved in Breast Cancer Cell Proliferation, Migration and Invasion

**DOI:** 10.3389/fonc.2021.799082

**Published:** 2022-01-04

**Authors:** Yanping Fan, Xiaotong Dong, Meizeng Li, Pengju Liu, Jie Zheng, Hongli Li, Yunxiang Zhang

**Affiliations:** ^1^ Pathology Department, First Affiliated Hospital of Weifang Medical University (Weifang People’s Hospital), Weifang, China; ^2^ Department of Basic Medicine, Weifang Medical University, Weifang, China; ^3^ School of Economics, Qingdao University, Qingdao, China

**Keywords:** breast cancer, long non-coding RNA, KRT19P3, PD-L1, CD8^+^ T cell, immune

## Abstract

Long non-coding RNAs (LncRNAs) have already been taken as critical regulatory molecules in breast carcinoma (BC). Besides, the progression of BC is closely associated with the immune system. However, the relationship between lncRNAs and the tumor immune system in BC has not been fully studied. LncRNA KRT19P3 has been reported to inhibit the progression of gastric cancer. In the present study, we first discovered that KRT19P3 was downregulated in BC tissues compared with para cancer tissue. Then we showed that KRT19P3 could be used as a marker to differentiate BC from para cancer tissue. Increased expression of KRT19P3 markedly inhibited the proliferation, migration, and invasion rate of BC cells *in vitro* and tumor growth of BC *in vivo*. Conversely, KRT19P3 knockdown by siRNA markedly promoted the proliferation, migration, and invasion rate of BC cells after being transfected. Comparison of clinical parameters showed an inverse relationship between the expression of KRT19P3 and pathological grade. Furthermore, immunohistochemistry (IHC) was applied to reveal the positive rate of the expression of Ki-67, programmed death-ligand 1 (PD-L1), and CD8 in BC tissues. Correlation analysis showed that Ki-67 and PD-L1 were inversely proportional to KRT19P3 but CD8 was directly proportional to KRT19P3. In conclusion, this study demonstrated that lncRNA KRT19P3 inhibits BC progression, and may affect the expression of PD-L1 in BC, which in turn affects CD8^+^ T (CD8 positive Cytotoxic T lymphocyte) cells in the immune microenvironment.

## Introduction

BC ranks the first in the diagnosis of female tumors and it is the fifth leading cause of death due to cancer worldwide (1–3). Despite advances in multiple types of treatments, the prognosis for BC patients remains dismal (4). For this reason, the identification of the mechanisms responsible for the pathogenesis of BC is urgent for improving the clinical outcome (5, 6).

LncRNAs are a kind of non-coding RNAs with methylguanosine cap and polyadenylate (poly-A) structure and a length of more than 200 nucleotides, most of which are transcripts of RNA polymerase II (7–9). At first, LncRNAs have been considered as non-functional genes. Nevertheless, with more deeply research, it has been discovered that lncRNAs play crucial roles in the progression of tumors by promoting or inhibiting biological behaviors such as cell proliferation, migration, and EMT (10–14), which allows tumor and other types of cells around the tumor microenvironment to interact with each other. Meanwhile, LncRNAs can regulate the expression of key genes associated with immune function affecting the function of immune cells involved in the microenvironment of tumors (15). For instance, lncRNA KCNQ1OT1 promoted the progression of prostate cancer by suppressing CD8^+^ T cells cytotoxicity through the KCNQ1OT1/miR-15a/PD-L1 axis (16). Yilong Ai et al. found that CRNDE specifically sponged miR-545-5p to induce T-cell immunoglobulin and mucin domain-3 (TIM-3), thus contributing to CD8^+^ T-cell exhaustion in Oral squamous cell carcinoma (OSCC) (17). Although the functional roles of lncRNAs in BC are diverse, the study of the relationship between lncRNAs and the tumor immune system is still in its infancy. T cell-regulated adaptive immune responses can induce the expression of PD-L1 in the tumor microenvironment (18). However, the expression of PD-L1 inhibits T lymphocytes from playing a role in the immune microenvironment (7). From this point of view, it is interesting that lncRNAs are involved in the immune process.

LncRNA KRT19P3 is a pseudogene located on Chromosome 4. It was reported to inhibit gastric cancer proliferation and invasion through the NF-κB signaling pathway (19). To study the role of KRT19P3 in BC, we have investigated the function of KRT19P3 in BC *in vitro* and its association with the immune. This study provides a theoretical and experimental basis for immune-related studies between lncRNA KRT19P3 and BC.

## Materials and Methods

### Cell Culture

BC cell line MDA-MB-231 was purchased from Procell Life Science & Technology Co., Ltd. (Wuhan, China). The cell line was cultured in RPM1640 medium (Solarbio, USA) added with 10% FBS (Hyclone, Logan, UT, USA). The environment in the incubator was maintained at 5% CO2 and a constant temperature of 37°C.

### Reverse Transcription-Quantitative Real-Time PCR

Axygen Total RNA Preparation Kit (Corning Life Sciences Co., Ltd., Suzhou, China) was used to extract total RNA. M-MuLV reverse transcriptase (New England Biolabs), RNase inhibitors, and dNTPs (Takara) was used to synthesize cDNA. A fluorescence quantitative PCR instrument (ABI7500Fast, USA) was used for RT-qPCR. GAPDH was used as an internal control.

The primer sequences are as follows:

GAPDH forward primer 5’-GCACCGTCAA-GGCTGAGAAC-3’;

Reversed primer 5’-TGGTGAAGACGCCAGTGGA-3’;

KRT19P3 forward primer5’-CAGTGAGAGGCAGAATCAGG-3’;

Reversed primer5’-TTGGAGGTGGACAGGCTATT-3’.

The relative expression of KRT19P3 was analyzed by 2^-△CT^.

### Cell Transfection

The pcDNA3.1-KRT19P3 plasmids and siRNAs were provided by Professor Jie Zheng, Department of Pathology, Weifang Medical College. Lipofectamine2000 (Invitrogen, USA) was used to transfect MDA-MB-231 cells. The plasmids and Lipofectamine 2000 Reagent were mixed and incubated in a 1: 1 ratio for 15 minutes and then added to a six-well plate. The siRNAs and Lipofectamine 2000 Reagent were mixed and incubated in a 1: 1.3 ratio for 15 minutes and then added to a six-well plate. The overexpression and knockdown efficiency was confirmed 24 h after transfection by qRT-PCR.

### EdU Assay

MDA-MB-231 proliferation capacity was detected by BeyoClick™ EdU-594 Cell Proliferation Assay Kit (Shanghai Biyuntian Biotechnology Co., Ltd., Shanghai, China). Cells after transfection 24 h were seeded onto 96-well plates, and three duplicate wells were set for each group, 2×10^4^ cells per well. Proliferation efficiency was the percentage of positive cells stained with EdU.

### Migration and Invasion Assay

The potential of invasion and migration was detected using transwell inserts (Corning, Cambridge, MA, USA). In the migration assay, 4×10^4^ cells were added above the chamber, RPMI1640 with 10% FBS in the lower chamber, and the chambers were fixed after 24 hours. Apodemsa staining solution (Solarbio, USA) was stained for 40 minutes and observed microscopically. Invasion assay was performed by adding Matrigel (Corning, Cambridge, MA, USA) on the top of the membrane to simulate cell transmembrane assay. We randomly took five fields for cell counting under the microscope.

### *In Vivo* Tumorigenic

Lentivirus vector LV-KRT19P3 and LV-NC transfected MDA-MB-231 cells. 1×10^7^ cells in 100μl of PBS were injected into the mammary fat pads of 4-week-old female athymic BALB/c nude mice (Vital River, Beijing, China). Mice were grouped (six for each group) and housed under SPF conditions at the Experimental Animal Center of Weifang Medical College. Tumor growth was observed weekly and tumor volumes (V) were calculated as V = (tumor length× width2)/2. Six weeks later, bioluminescence was used in the IVIS system. These mice were killed and tumor nodules were extracted and evaluated with hematoxylin and eosin staining. Animal use in the study was performed following the animal care and ethical committee of the medical sector in Weifang Medical College.

### Human tissues

A total of 98 BC tissues and non-tumor breast tissues 5cm away from the cancer region were obtained from the First Affiliated Hospital of Weifang Medical University from Feb 2019 to Jun 2019. Half of the tumor tissues were paraffin-embedded, and the other half tissues were stored at -150°C for RT-qPCR detection. The above specimens have been diagnosed as BC by clinicians. This study conformed to the standards of the Ethics Committee of Weifang People’s Hospital.

### Immunohistochemistry

The expression of CD8^+^ T and PD-L1 genes in 80 breast cancer tissues and matching normal tissue specimens were detected by IHC. Xylene was used to deparaffinized and diluted graded alcohols to hydrated; pH 9.0 citric acid buffer was used to repair antigens at high temperature and pressure; endogenous peroxidase was blocked with 0.3% H_2_O_2_; and incubated with primary antibodies of CD8 and PD-L1 (Beijing Zhongshan Jinqiao, China) for an hour at room temperature. Finally, DAB for color staining. The positive side of CD8 protein staining was mainly localized on the nucleus, and the experimental results were judged according to the proportion of positive cells, with 10% as the cutoff value, ≥ 10% as positive, and<10% as negative.

### Statistical Analysis

SPSS 25 software was used for statistical analysis. Graph Pad Prism7 software was used for picture drawing. We used ROC (receiver operating curve) to assess the diagnostic value of KRT19P3 in BC and normal breast. Chi-square test and Fisher’s exact test were used to analyze the relationship between KRT19P3 and clinical parameters as well as PD-L1. The correlation analysis was performed using Spearman’s correlation test. Differences between the two groups were analyzed using a T-test. *P* value<0.05 was considered statistically significant.

## Results

### LncRNA KRT19P3 Inhibited BC Cell Proliferation, Migration, and Invasion *In Vitro*

**Figure 1:**
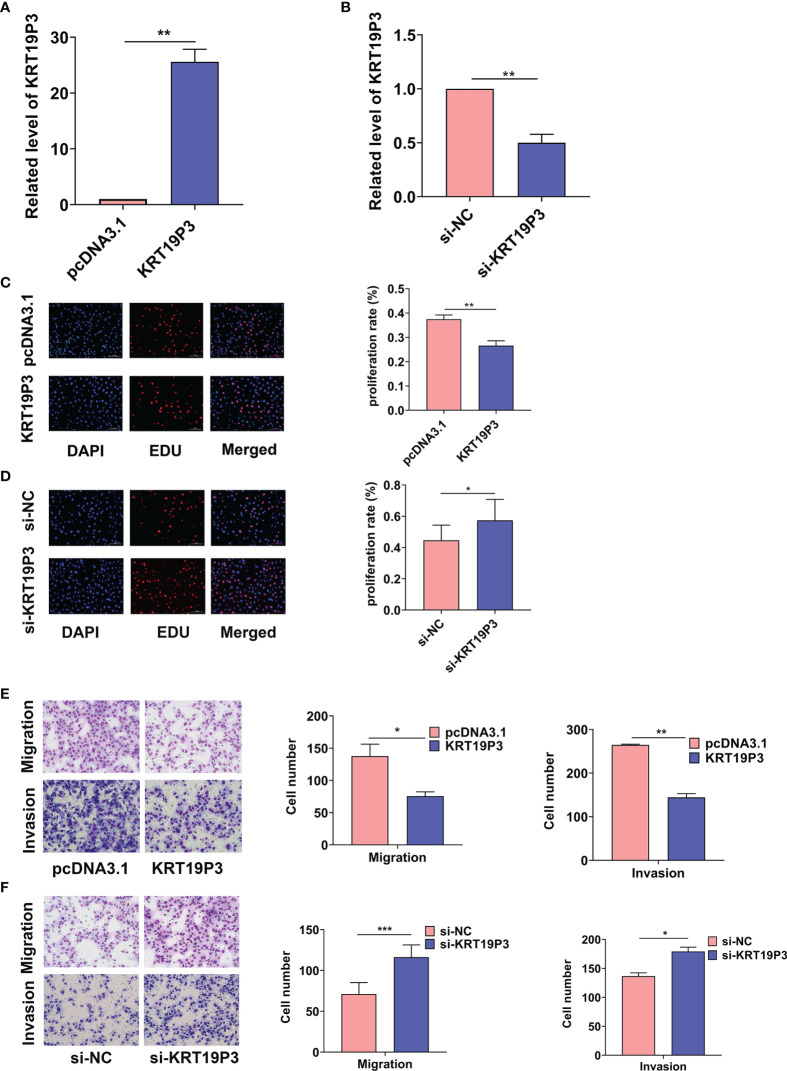
KRT19P3 inhibited BC cell proliferation, migration and invasion. **(A)** RT-qPCR results showed that pcDNA3.1-KRT19P3 transfection significantly increased the KRT19P3 level in MDA-MB-231 cells. **(B)** RT-qPCR results showed that siRNA transfection significantly decreased the KRT19P3 level in MDA-MB-231 cells. **(C)** EDU assay to detect the effect of overexpression of KRT19P3 on cell proliferation. **(D)** EDU assay to detect the effect of interfering KRT19P3 on cell proliferation. **(E)** Transwell assay to detect the effect of overexpression of KRT19P3 on cell migration and invasion. **(F)** Transwell assay to detect the effect of interfering with KRT19P3 on cell migration and invasion. *P < 0.05, **P < 0.01, ***P < 0.001.

Regarding the KRT19P3 played an important part in gastric cancer cell migration and invasion capability, we investigated the role of KRT19P3 on BC cells. To reveal the role of KRT19P3 in BC cell proliferation, we overexpressed KRT19P3 with pcDNA3.1(+) plasmid vector or downregulated KRT19P3 using siRNA in MDA-MB-231 cells. The overexpression and knockdown efficiency was confirmed by qRT-PCR, with 24.6 ± 2.3-fold increase and 0.5 ± 0.1-fold decreases respectively, in MDA-MB-231 cells after 24 h transfection (**Figures 1A, B**). EdU assays were carried out to examine cell proliferation ability in different groups. The results showed that overexpression of KRT19P3 significantly inhibited MDA-MB-231 cells proliferation by 10.85% (**Figure 1C**), whereas, KRT19P3 knockdown enhanced cell proliferation by 12.79% compared with a control group (**Figure 1D**). In addition, the transwell assay revealed that upregulation of KRT19P3 substantially increased the migration and invasion rate of MBA-MD-231 cells by 45.13% and 45.45% respectively (**Figure 1E**). In contrast, KRT19P3 knockdown remarkably decreased the migration and invasion rate of MBA-MD-231 cells by 38.85% and 23.69% respectively (**Figure 1F**). In conclusion, the results showed that KRT19P3 functions as a tumor suppressor gene *in vitro*. These results indicated that KRT19P3 suppressed tumorigenesis *via* reducing proliferation capacity, migration, and invasion abilities of BC cells *in vitro*.

### LncRNA KRT19P3 Inhibited BC Growth *In Vivo*

**Figure 2:**
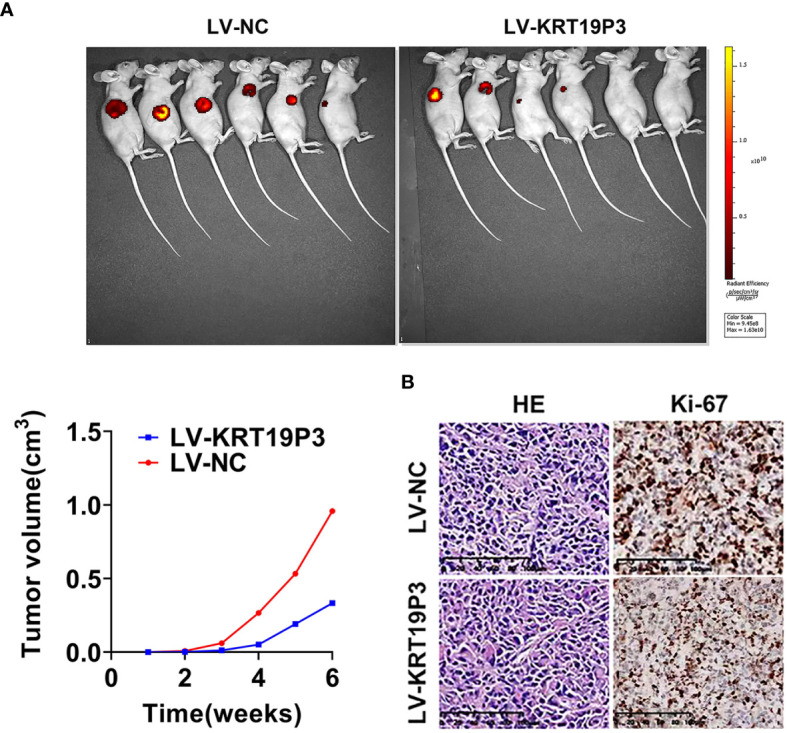
LncRNA KRT19P3 inhibited BC growth *in vivo.* **(A)** A stable MDA-MB-231 cell line overexpressing KRT19P3(LV-KRT19P3) was constructed and injected under the subcutaneous of nude mice to establish a xenograft tumor model with 6 mice in each group. Moreover, primary tumor growth was measured every week after injection. The tumor volumes in the LV-KRT19P3 group were significantly smaller than those in the LV-NC group. **(B)** Immunohistochemistry for Ki-67 detection revealed that cancer cells in the LV-NC group showed a higher positivity rate than those in the LV-KRT19P3 group (magnification × 200).

Vitro experiments showed that KRT19P3 functions as a tumor suppressor gene. We further explored the effect of KRT19P3 on BC cells *in vivo*. The results showed that the tumor volumes of LV-KRT19P3 transfected cells were significantly smaller than that of LV-NC transfected xenograft tumors (**Figure 2A**). Immunohistochemistry for Ki-67 detection revealed that tumor cells in the LV-KRT19P3 group showed a lower positivity rate than those in the LV-NC group (**Figure 2B**). In summary, these data demonstrated that KRT19P3 suppressed BC *in vivo*.

### LncRNA KRT19P3 Significantly Decreased in Human BC Tissues

KRT19P3 inhibits the functions of BC cells through the results *in vitro* and *in vivo*. We explored the relationship between KRT19P3 and clinical parameters.

**Figure 3:**
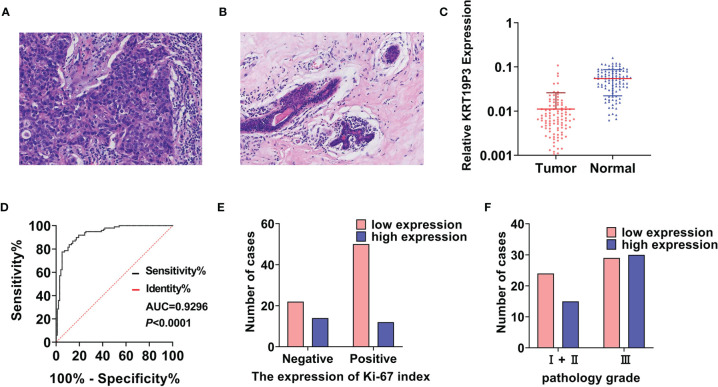
LncRNA KRT19P3 significantly decreased in human BC tissues. **(A)** HE pictures of breast cancer tissue (H&E × 200). **(B)** HE pictures of normal breast tissue (H&E × 200). **(C)** The expression of KRT19P3 in human breast cancer tissues (Tumor, n=98) and corresponding para cancer tissues (Normal, n=98) was detected by RT-qPCR. **(D)** The ROC curve was drawn according to the expression of KRT19P3 in breast cancer tissues and para cancer tissues (AUC=0.9296, *P*<0.0001). **(E)** Relationship between KRT19P3 and Ki-67 index (Ki-67 positive rate ≥30%, r= -0.213, *P*<0.05). **(F)** Relationship between KRT19P3 and pathology grade (r= -0.227, *P*<0.05).

Paired specimens were collected from 98 pairs of female BC patients (**Figures 3A, B**), and the tumor size ranged from 0.60 cm to 9.00 cm. The RT-qPCR result showed that the median expression of KRT19P3 was 0.0063 in BC tissues and 0.0550 in non-tumor tissues. KRT19P3 was 8.7302-fold higher in para cancer tissues than in tumor tissues (**Figure 3C**). The ROC curve was drawn according to the expression of KRT19P3 in BC and para cancer tissues and the AUC under the curves was calculated. AUC was 0.9296, indicating that KRT19P3 could better differentiate BC tissues from non-tumor tissues (**Figure 3D**).

Moreover, IHC results indicated that Ki-67 expression was reduced in the KRT19P3 up-regulated group compared with the control group (**Figure 3E**). On the other hand, a comparison of clinical parameters showed an inverse relationship between KRT19P3 and pathological grade (**Figure 3F**).

### LncRNA KRT19P3 Correlated With PD-L1 and CD8^+^ T Cell

**Figure 4:**
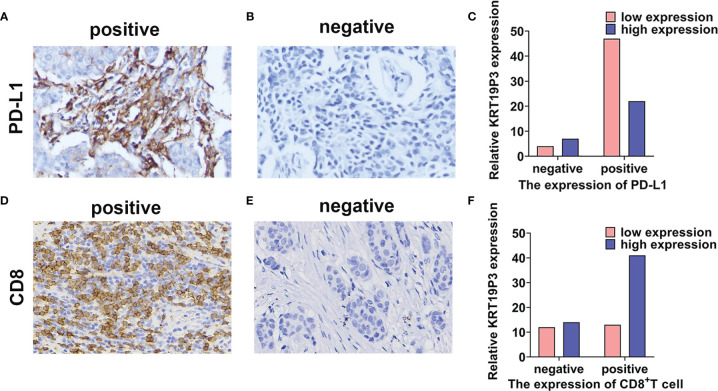
LncRNA KRT19P3 correlated with PD-L1 and CD8^+^ T cell. **(A, B)** PD-L1 expression in breast cancer tissue (**A** positive staining, 200x; B negative staining, 200x). **(C)** KRT19P3 expression was lower in the PD-L1-positive group than in the PD-L1-negative group in breast cancer tissues (n=80). **(D, E)** CD8 expression in breast cancer tissues (**D** positive staining, 200x, **E** negative staining, 200x). **(F)** KRT19P3 expression was higher in the CD8^+^ T high group than in the CD8^+^ T cell low expression group in breast cancer tissues (n=80).

We conducted IHC experiments to detect the positive rate of PD-L1 and CD8^+^ T in tissues from 80 pairs of breast cancer patients. Statistical analysis indicated that the expression of PD-L1 was significantly lower (**Figures 4A–C**) and CD8^+^ T was significantly higher (**Figures 4D–F**) in the KRT19P3 high expression group. Spearman analysis revealed that KRT19P3 is negatively correlated with PD-L1 (*r*= -0.227, *P*=0.047) and positively correlated with CD8^+^ T (*r*= 0.223, *P*= 0.046, **Table 1**). The results suggested that KRT19P3 may affect the function of CD8^+^T through the PD-1/PD-L1 axis.

**Table 1:** The relationship between KRT19P3 and PD-L1, CD8 in BC tissues.

	Number	KRT19P3		χ^2^	P-value
		Low expression	High expression		
PD-L1				4. 139	0. 047*
Negative	11	4	7		
Positive	69	47	22		
CD8				3. 982	0. 046*
Negative	26	12	14		
Positive	54	13	41		

*P < 0.05.

## Discussion

Oncogenesis is a gradual process, involving multistage reactions and the accumulation of multiple molecular mutations. LncRNAs have been identified as critical players in oncogenesis and immune response. However, the process of lncRNA involvement in tumor immunity remains incompletely elaborated. KRT19P3, located on chromosome 4, is a transcriptional length of 828 bp of lncRNA, suggesting that it can participate in a variety of biological processes (20).

It was confirmed that KRT19P3 was highly expressed in bladder cancer tissues compared to normal bladder tissues (21). KRT19P3 was less expressed in gastric cancer tissues compared to normal gastric tissues (19). However, the role played by KRT19P3 in the BC is unclear. It is well known that proliferation, migration, and invasion are the obvious biological features of malignant tumors (22). In the present study, we provide evidence for a functional role of lncRNA KRT19P3 in cell proliferation, migration, and invasion of BC. We found that overexpression of KRT19P3 inhibited the proliferation, migration, and invasion of BC cells *in vitro* and the growth of xenografts in nude mice. Taken together, KRT19P3 may function as an

Subsequently, we confirmed the differential expression of KRT19P3 in breast cancer tissues and para cancer tissues by clinical samples. The expression of KRT19P3 was higher in non-tumor tissues than in BC tissues. The expression of KRT19P3 in BC tissues and para cancer tissues was plotted by the ROC curve. The result showed that AUC under the curve was 0.9626, indicating that KRT19P3 could be used as a better index to distinguish BC from normal breast tissues. Correlation analysis with clinical parameters showed that the expression of KRT19P3 in the high Ki-67 index group was lower than that in the low Ki-67 index group. It is well known that Ki-67 is an important clinical proliferative marker for many types of cancer, and a high Ki-67 index predicts a poor prognosis for patients (23). Qiu et al. found that the expression of oncogene LINC00668 was positively correlated with the Ki-67 proliferation index in BC tissues, and showed by cell function experiments that downregulation of LINC00668 expression decreased the proliferation of BC cells (24). Similarly, the expression of KRT19P3 in BC tissues was inversely correlated with pathological grade, which is consistent with the finding of a significant positive correlation between the expression of the oncogene lncRNA GClnc1 and histological grade in bladder cancer tissues found by Zhuang et al. (25). Comprehensive analysis showed that KRT19P3 could inhibit the progression of BC, which further verified the results of *in vitro* cell experiments and *in vivo* animal experiments. Meanwhile, the expression of KRT19P3 in BC tissues correlated with the PD-L1 and CD8^+^ T cells. The results showed that in the group with high KRT19P3 expression, the PD-L1 positivity rate decreased while the CD8^+^ T cell positivity rate increased. the expression of KRT19P3 was negatively correlated with PD-L1 and positively correlated with CD8+ T cells.

The tumor immune microenvironment is the internal environment for tumors development and metastasis (13, 16, 26, 27). CD8^+^ T cells play an important role in the elimination of tumor cells and their continued proliferation and growth after the elimination of tumor immune escape (28) and are also the main effector cells in the elimination of tumor cells in the internal environment (29). However, PD-L1 is a protein molecule that inhibits the action of effector T cells and is one of the ligands of PD-1, which is expressed by a variety of tumor cells as well as immune cells. High expression of PD-L1 in BC is associated with prognostic markers of malignancy (30, 31). When PD-1 binds to its receptor, PD-L1, it transmits negative regulatory signals to T cells and induces them to become dormant. Based on the correlation analysis between KRT19P3 and PD-L1, CD8^+^ T, it was hypothesized that KRT19P3 may inhibit BC progression by reducing PD-L1 expression in tumor cells and activating the tumor-killing potential of CD8^+^ T cells, which is also consistent with the findings of Sun et al. (32), who blocked the PD-1/PD-L1 pathway and thus rescued the depleted CD8^+^ T cells.

In summary, our study confirmed that KRT19P3 was less expressed in BC than in normal breast tissues and inhibited the function of BC cells. Combined with clinical parameters, KRT19P3 played the role of the tumor suppressor gene in BC. The expression of KRT19P3 was negatively correlated with PD-L1 but positively correlated with CD8^+^ T cells. Therefore, KRT19P3 may inhibit BC progression through the immune pathway. We will actively explore the specific mechanism in the follow-up study. This finding may provide a new direction for the diagnosis and treatment of BC.

## Data Availability Statement

The original contributions presented in the study can be directed to the corresponding author.

## Ethics Statement

The animal study was reviewed and approved by Ethics Committee of Weifang People’s Hospital.

## Author Contributions

YZ: guarantor of integrity of the entire study, study concepts, and manuscript editing. YF, XD, and ML: study design, manuscript review, clinical sample collection, experimental procedures, and statistical analysis. PL: English Language Embellishment. JZ and HL: resources, laboratory samples, and instrumentation supplied. All authors read and approved the final manuscript.

## Conflict of Interest

The authors declare that the research was conducted in the absence of any commercial or financial relationships that could be construed as a potential conflict of interest.

## Publisher’s Note

All claims expressed in this article are solely those of the authors and do not necessarily represent those of their affiliated organizations, or those of the publisher, the editors and the reviewers. Any product that may be evaluated in this article, or claim that may be made by its manufacturer, is not guaranteed or endorsed by the publisher.
